# Supplementary material for: The Umbelopsis ramanniana Sensu Lato Consists of Five Cryptic Species
Source: J Fungi (Basel). 2022 Aug 23;8(9):895. doi: 10.3390/jof8090895 (PMC9506118; doi:10.3390/jof8090895)

Figure S1. Phylogenetic tree based on nLSU rDNA D1/D2 region inferred from neighbor-joining (NJ) analysis for *Umbelopsis ramanniana* and related species. Branches with bootstrap values ≥ 50% were given. The taxonomic position of the strains was indicated in parentheses. "Subclade" following *U. ramanniana* strains were the treatments by Ogawa et al [28], while those new species were established in this study. T = ex-type strain, IT = ex-isotype strain and HT = ex- holotype strain.

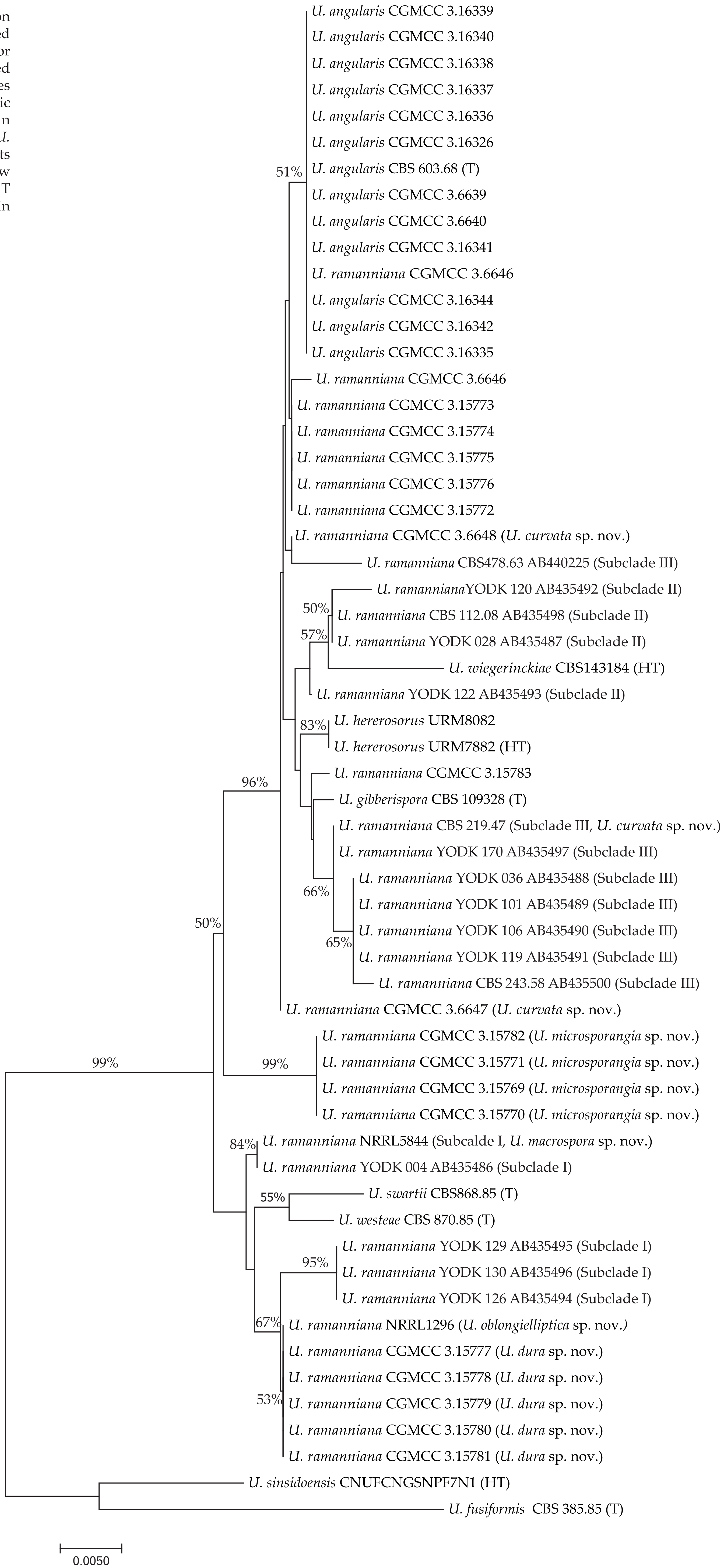

Supplement: Supplementary file 1 [file jof-08-00895-s001.zip › jof-1863219-supplementary/Figure S1.pdf]
