# Supplementary material for: The Umbelopsis ramanniana Sensu Lato Consists of Five Cryptic Species
Source: J Fungi (Basel). 2022 Aug 23;8(9):895. doi: 10.3390/jof8090895 (PMC9506118; doi:10.3390/jof8090895)
Supplement: Supplementary file 1 [file jof-08-00895-s001.zip › jof-1863219-supplementary/Table S2.pdf]

Table S2. Maximum growth temperature (°C) of strains in the *Umbelopsis ramanniana* complex.

**C1: *U. curvata* [35–36]**					
CGMCC 3.6647	35	CGMCC 3.6648	36	CBS 219.47	35
**C2: *U. ramanniana* [33–35 (–36)]**					
CGMCC 3.6646	33	CGMCC 3.15772	33	CGMCC 3.15773	33
CGMCC 3.15774	33	CGMCC 3.15775	33	CGMCC 3.15776	33
CGMCC 3.15783	36	CGMCC 3.15784	34	CGMCC 3.15785	35
CGMCC 3.15786	35	CGMCC 3.16356	35		
**C3: *U. microsporangia* [31–32]**					
CGMCC 3.15769	31	CGMCC 3.15770	32	CGMCC 3.15771	32
CGMCC 3.15782	32				
**C4: *U. dura* [35]**					
CGMCC 3.15777	35	CGMCC 3.15778	35	CGMCC 3.15779	35
CGMCC 3.15780	35	CGMCC 3.15781	35		
**C5: *U. oblongielliptica* [37]**					
NRRL 1296	37				
**C6: *U. macrospora* [38]**					
NRRL 5844	38				
